# Microalgae Biomass Production from Rice Husk as Alternative Media Cultivation and Extraction of Phycocyanin Using 3D-Printed Ohmic Heating Reactor

**DOI:** 10.3390/foods13091421

**Published:** 2024-05-06

**Authors:** Gabriela Cid-Ibarra, Rosa M. Rodríguez-Jasso, Gilver Rosero-Chasoy, Ruth Belmares, Juan Carlos Contreras-Esquivel, Samanta Machado-Cepeda, Alejandra Cabello-Galindo, Héctor A. Ruiz

**Affiliations:** 1 Biorefinery Group, Food Research Department, School of Chemistry, Autonomous University of Coahuila, Saltillo 25280, Coahuila, Mexicorrodriguezjasso@uadec.edu.mx (R.M.R.-J.); samanta.machado@uadec.edu.mx (S.M.-C.);; 2 Functional Foods and Nutrition Group, Food Research Department, School of Chemistry, Autonomous University of Coahuila, Saltillo 25280, Coahuila, Mexico; ruthbelmares@uadec.edu.mx (R.B.);; 3 Laboratory of Applied Glycobiotechnology, Food Research Department, School of Chemistry, Autonomous University of Coahuila, Saltillo 25280, Coahuila, Mexico

**Keywords:** biorefinery, *Spirulina platensis*, pigment, bioeconomy, high added-value compounds, fermentation

## Abstract

Phycocyanin is a highly valued pigment present in *Spirulina platensis* biomass with applications in the food industry in terms of biorefinery concepts; specifically, its antioxidant and antimicrobial capacity are an advantage that could be incorporated into a food matrix. This study aims to use rice husk as an alternative culture medium for *S. platensis* biomass growth and phycocyanin extraction by ohmic heating processing using a 3D-printed reactor. *S. platensis* was cultivated in rice husk extract (RHE) from 0–100% (*v*/*v*). The highest content of microalgal biomass was 1.75 ± 0.01 g/L, with a specific growth rate of 0.125 ± 0.01 h^−1^. For the phycocyanin extraction under an ohmic heating process, a 3D-printed reactor was designed and built. To optimize phycocyanin extraction, a central composite rotatable design (CCDR) was evaluated, with three factors: time (min), temperature (°C), and pH. The highest phycocyanin content was 75.80 ± 0.98 mg/g in *S. platensis* biomass grown with rice husk extract. Ohmic heating is a promising method for rapid phycocyanin extraction, and rice husk as a culture medium is an alternative for the growth of *S. platensis* biomass in the integration of second- and third-generation biorefineries.

## 1. Introduction

The intensity of color is a crucial aspect when it comes to the appearance of food products, and it plays a significant role in consumer perception. This makes it attractive to the consumer and increases their potential to choose a particular product, hence its economic relevance [1,2]. Artificial colorants are used because they are cheap, stable, and bright; however, health complications are found when they are consumed in a food matrix [3]; some of the possible complications are allergies and neurological disorders. Synthetic dyes have been restricted and investigated by the U.S. Food and Drug Administration (FDA) and California Office of Environmental Health Hazard Assessment (OEHHA), as well as the European Food Safety Authority (EFSA) [4,5,6,7].

For this reason, natural dyes are gaining interest, as they are not harmful and have no undesirable or adverse effects on human health. Natural pigments can be beneficial due to their bioactive compounds [8,9]. Among natural dyes, microalgal biomass can be used as a natural resource to obtain interesting pigments. Some intracellular pigments of industrial interest in microalgae biomass are chlorophylls, carotenoids, xanthophylls, and phycobiliproteins (phycocyanin) [10]. Phycocyanin is part of a complex protein pigment called a phycobiliprotein (phycoerythrin, phycocyanin, and allophycocyanin), which gives a blue-green color to the microalgal biomass, and this pigment represents about 40–60% of the total protein content of the biomass [11]. These pigments are found in aquatic organisms such *Acanthophora specifera*, *Bangia atropurea*, *Gracilaria corticata*, *Laurencia papillosa*, and *S. platensis* [12,13], and due to the phycocyanin content in these microorganisms, they have a blue-green color.

Phycocyanin is used extensively in the pharmaceutical and food industries due to its antioxidant, anti-inflammatory, and anti-microbial properties. Food-grade phycocyanin costs approximately USD 0.13 per milligram, and analytical grade phycocyanin costs USD 15.00 per milligram [14]. Due to its widespread application, phycocyanin has generated USD 250 million in the last decade [15]. Some examples of phycocyanin use in the food industry include ice cream, beverages, and confectionery [13]. One of the biggest challenges in the obtention of phycocyanin is the extraction process; the most common methods to hydrolyze or break down the cell wall and release the pigment are freezing and thawing cycles, bead milling, ultra-sonication, and ohmic heating [16,17,18].

**Figure 1:**
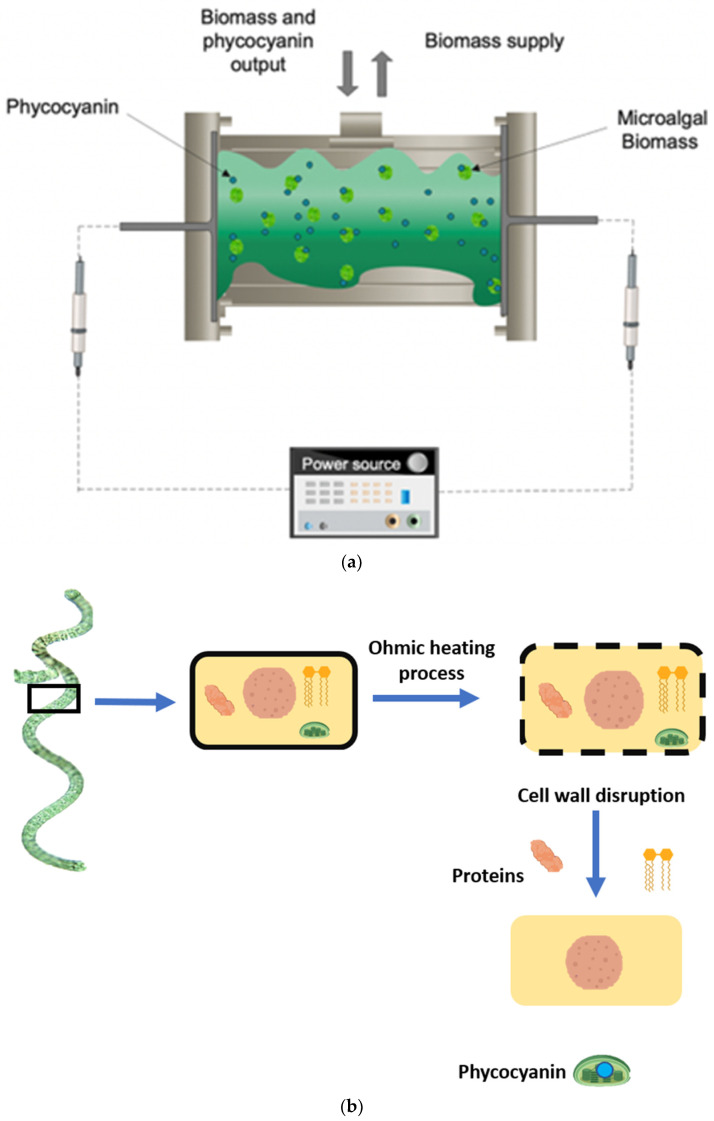
(**a**) Ohmic heating reactor. (**b**) Diagram of cell wall disruption by ohmic heating process.

Among the various methods for disrupting cell wall of *S. platensis*, freezing and thawing have been reported to be the highest-yielding methods for obtaining phycobiliproteins [19]. However, the cycles of freezing and thawing may affect the stability of the compounds from the aggressive cell wall disruption. Another alternative is the ohmic heating process, in which an electrical field is applied to a conductive sample [9]. In the case of cellular material, such as *S. platensis*, ohmic heating causes electroporation of the cell membrane. This occurs when the electrical field applied in the sample has a higher dielectric strength, and the lipids present in the membrane are disrupted, leading to permeabilization of the membrane and releasing intracellular compounds such as phycocyanin [20]. Figure 1 shows the mechanisms and effects of ohmic heating processing on *S. platensis*. Ohmic heating has disadvantages, including possible corrosion of the electrodes and the complex conductivity of the sample [21]. Nevertheless, ohmic heating offers interesting advantages such as short time of extraction and homogeneity of heating [10]. In addition, ohmic heating allows the implementation and design of a reactor, depending on the requirements of the sample, the operational conditions, and the possibility of using different materials for construction.

These characteristics lead to ohmic heating being an alternative method for the extraction of phycocyanin. The phycocyanin present in *S. platensis* is close to 20% of the total biomass [22]; however, this extraction process gives a low yield of these pigments. Although ohmic heating is an interesting alternative for phycocyanin extraction, various conditions need to be studied in detail to achieve better yields of phycocyanin using the method. Factors such as temperature, time, and pH must be studied in detail.

In order to implement an extraction method for phycocyanin, improvements in the cultivation of *S. platensis* have been sought; some desirable characteristics for the growth of a culture are higher yield, ease of implementation and operation, and time of culture growth. An alternative growth medium involves the use of agro-waste [23]. Some examples are tofu wastewater [24], cheese whey [25], and fish waste [26]. A viable alternative is the use of rice husk to grow *S. platensis* for the subsequent extraction of phycocyanin. Rice husk is a residue produced from rice milling [27] and accounts for about 20% of the whole rice grain. This agro-waste is used to produce biofuels, livestock feed, and bio-fertilizers [28]. Due to the composition of rice husk, it can be used for the growth of photosynthetic organisms [29]. The main components of rice husk compounds are SiO_2_, SiC, Si_3_N_4_, Si, Mg_2_Si, hemicellulose 24.3%, cellulose 34.4%, lignin 19.2%, starch, and protein [30]. To date, the utilization of rice husk extract as a culture medium has not been explored for the growth of *S. platensis*; additionally, the starch content and trace metals present in the waste can be used for such growth. This work aims to produce *S. platensis* biomass from rice husk as an alternative for cultivation and to extract phycocyanin using a 3D-printed ohmic heating reactor in terms of a biorefinery concept.

## 2. Materials and Methods

### 2.1. Pre-Inoculum and Growth Conditions

*S. platensis* LEB-52 strain was kindly provided by the Passo Fundo University, Brazil (Prof. Luciane Colla). *S. platensis* was cultivated in previously sterilized Zarrouk medium [31]. The growth conditions for the pre-inoculum were photo periods of 13 h of light and 11 h of darkness, a light intensity of 73 μmol/(m^2^×), and room temperature for 10 days. The working volume was 250 mL in an Erlenmeyer flask with a capacity of 500 mL [31].

### 2.2. Rice Husk Extract as a Cultivation Medium

The rice husk (RH) was obtained from Gea (Jalisco, Mexico). The water-soluble compounds present in the rice husk were extracted. Briefly, 40 g of rice husk was weighed and placed in a Schott flask to which 1 L of distilled water was added. The extraction of rice husk components was carried out for 1 h in a water bath (100 °C) at an internal temperature of 76 °C (Schott flask). After the extraction step, the solids from the rice husk were removed by filter paper using a vacuum pump, and the liquid extract of the rice husk was sterilized with UV light for 15 min. The final pH of the rice husk extract was 7.5, and the rice husk was stored at 4 °C until use [32].

### 2.3. Experimental Setup for the Growth of S. platensis Using Rice Husk as a Culture Medium

The *S. platensis* growth was evaluated in the rice husk extract at different concentrations. Table 1 shows the different treatments to determine the effect of rice husk extract on the growth of *S. platensis*. All experiments were batch-run with an inoculum content of 15% (*v*/*v*). All the treatments were sterilized with UV light for 15 min prior to inoculation. The working volume was 300 mL in an Erlenmeyer flask of 500 mL, and the experimental conditions were the same as mentioned above; all treatments had a constant nitrogen source of 0.7 g/L NaNO_3_. The treatments T1–T6 contained different percentages of rice husk extract (0–100%) supplemented with a nitrogen source. The control (T7) was Zarrouk medium.

The sampling was carried out for 2 days during the growth kinetics over 14 days. A sample of 10 mL was taken to determine the biomass concentration. The protein and phycocyanin contents were evaluated at the end of the period [33]. For microalgal kinetics, the phase of highest yield, known as the exponential phase, was used, in which the highest amount of biomass produced (g/L) is related to time (days) in an exponential function [34].

### 2.4. Analytical Methods for the Characterization of Rice Husk Extract and S. platensis Biomass

#### 2.4.1. Carbohydrate Determination in Rice Husk Extract

The carbohydrate content in the rice husk extract (RHE) was determined using the anthrone method. To prepare the anthrone reagent, 0.2 g of anthrone was dissolved in 5 mL of 96% ethanol and 75% H_2_SO_4_. For the total carbohydrate assay, 200 µL of rice husk extract was mixed with 800 µL of the anthrone reagent in a test tube. A glucose solution (1000 ppm) was used in the calibration curve for a standard solution. The samples were vortexed and heated at 100 °C for 5 min, followed by cooling for 5 min. Then, 200 µL of each sample was transferred to a microplate and measured using a spectrophotometer (SunriseTM A-5082, Grödig, Austria) at 530 nm [31].

#### 2.4.2. Fourier-Transform Infrared Spectroscopy (FTIR) for the Analysis of Rice Husk and Phycocyanin

For the identification of functional groups from starch present in the rice husk extract an FTIR analysis was performed; starch can be used for the growth of microalgae as a carbon source. The rice husk (RH) and the rice husk extract (RHE) (lyophilized) were ground, sieved, and analyzed by FTIR using a Perkin Elmer Frontier FTIR spectrometer (Waltham, MA, USA) under the following conditions: resolution at 4 cm^−1^ with 16 scans per test. Spectra were collected from 4000 to 500 cm^−1^ in absorbance mode, normalized, and baseline corrected [34].

#### 2.4.3. Elemental Characterization by X-ray Fluorescence of Rice Husk

For determination of the elemental composition of the rice husk extract (RHE), milling and subsequent screening of the husk were performed, while to obtain the solids contained in the rice husk extract, lyophilization was used (Labconco company, Kansas City, MO, USA). The resulting solid was weighed (0.2 g) and analyzed by X-ray fluorescence (Panalytical, Epsilon 1, Almelo, The Netherlands), before being integrated by a spectrometer with the use of the Omniam software [35]. For the total solids content in the rice husk extract, the liquid part was evaporated, and the rest of the solids were weighed and heated at 600 °C for 3 h for the de-ashed content.

#### 2.4.4. Characterization of the Rice Husk by HPLC

For the characterization of RHE, high-performance liquid chromatography (HPLC) was performed. For hydrolysis, 0.5 g of the rice husk was weighed (previously ground and sifted) and placed in a flask to which 5 mL of H_2_SO_4_ at 72% was added. The samples were agitated manually for 1 h at 30 °C. After the agitation of the samples, water was added to dilute the acid to 4%. Post-hydrolysis, the sample content in the flasks was autoclaved for 1 h at 121 °C. After the treatment, the samples were filtered, and the solid part of the samples was dried for 24 h and weighed for the determination of the amount of Klason lignin present in the rice husk. The liquid phase of the sample was filtered through a 0.45 µm nylon filter for the injection of the sample into the HPLC system (Agilent 1260 Infinity II, Ratingen, Germany). The analysis was performed on Metacarb 87H (300 mm × 7.8 mm, Agilent, Ratingen, Germany) at temperature of 60 °C with a 0.7 mL/min flow rate of 0.005 mol/L sulfuric acid as the mobile phase. Xylose, arabinose, glucose, and acetic acid were determined and related to the polysaccharides present in the rice husk [36].

### 2.5. Determination of the Biomass Growth S. platensis

A representative sample of the culture medium (10 mL) was filtered using vacuum filtration, and the filter with the biomass was dried at 50 °C for 24 h. Subsequently, the biomass (content in the filter) was weighed to determine the dry weight of the biomass. During the 14-day growth period, a sample was taken every 2 days to obtain the growth kinetics [37].

### 2.6. Determination of Protein Content in Microalgal Biomass

For the release of the proteins present in the *S. platensis* biomass, the freezing and thawing method in three cycles was applied for cell wall breaking; after this step, the biomass (10 mL) was centrifuged at 6000 rpm for 15 min at 0 °C. After the centrifugation, the supernatant was discarded, and the pellet (biomass) was mixed with 1.5 mL of 25% (*v*/*v*) ethanol in NaOH (1 N). The samples were centrifuged again and sonicated for 15 min (110 W). For the quantification of proteins, 100 µL of the samples was used and placed in a microplate, and a standard curve was elaborated using bovine serum albumin; the regents of the Lowry method [38,39] were used, and the analysis required the use of the colorimetric method in a microplate spectrophotometer (Sunrise M A-5082, Grödig, Austria) at 660 nm.

#### Determination of Phycocyanin Content

A culture volume of 45 mL of the microalgal biomass was harvested at 14 days. The harvested sample was centrifugated at 6000 rpm for 15 min. The liquid phase was discarded, and the pellet was re-suspended with a phosphate buffer at pH 6.6 [16]. The pellet with buffer was mixed by vortexing. To evaluate the phycocyanin content present in the *S. platensis* biomass from the different treatments of rice husk extract, the samples were subjected to five cycles of freezing and thawing as an extraction method to release the intracellular phycocyanin. The phycocyanin was separated from the biomass by decantation. The phycocyanin concentration was determined by absorbance at 620 and 652 nm. The phycocyanin concentration in mg/mL was calculated from Equation (1) [16].

CPC = (OD620 − 0.47OD 652)/5.34(1)

where CPC is the C-phycocyanin concentration in mg/mL and OD is the optical density, reading at 620 and 652 nm. The remaining values correspond to constants for phycocyanin.

### 2.7. Design of Ohmic 3D Reactor for the Phycocyanin Extraction

For the extraction of phycocyanin from *S. platensis*, the ohmic heating processing technology was used, and a 3D-printed reactor was designed and built. The software employed for this purpose was Solidworks 2018, the software provided an effective means of design and development of images, and these files are compatible with the 3D printer.

**Figure 2:**
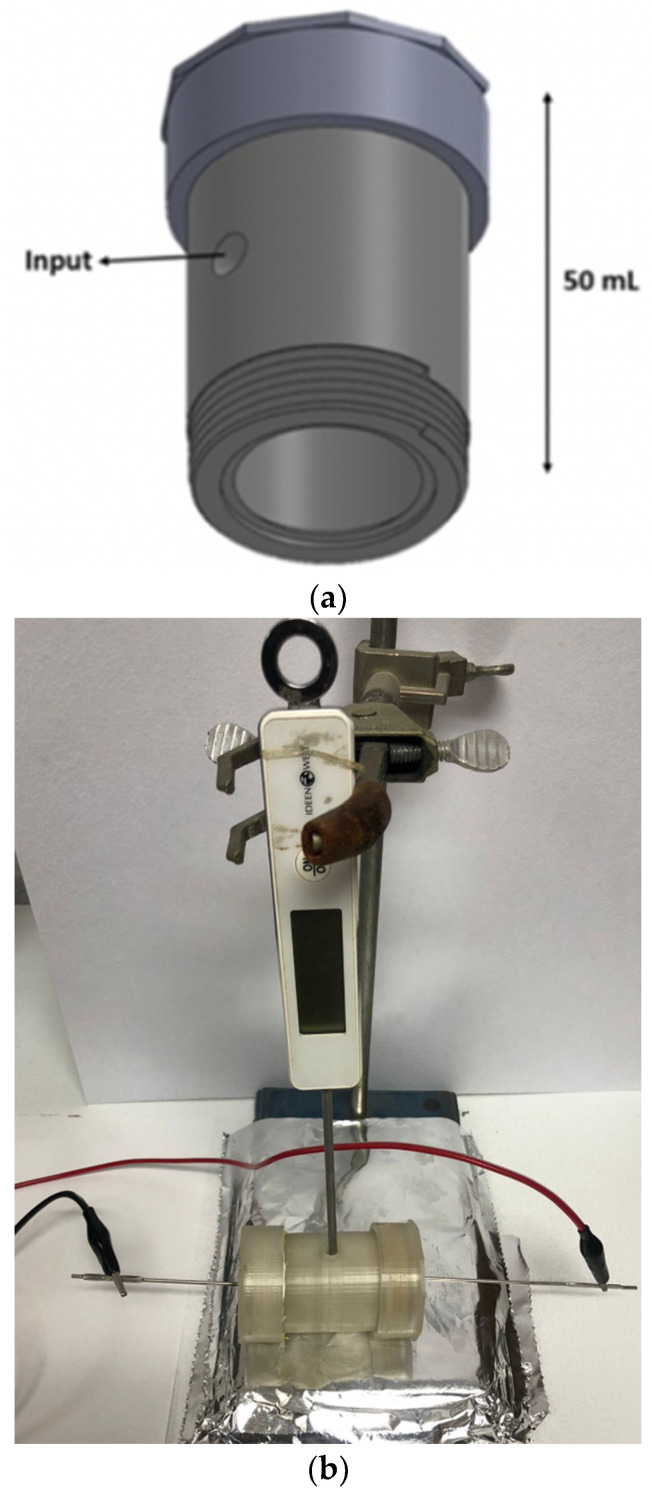
(**a**) 3D reactor for ohmic heating processing. (**b**) Photo of the ohmic heating reactor.

The design of the reactor was created with the following dimensions: 15 mm of radium, 50 mm of height, 6 mm of thickness, and a nominal volume of 50 mL. The reactor also has a hole in the middle with a radius of 3.5 mm. The reactor has two caps made of polylactic acid with the following dimensions: 21 mm radius, 15 mm of height, 3 mm of thickness, and a hole in the middle of each cap with a 3.5 mm radius. Figure 2 shows the reactor design. The reactor was printed on a 3D printer (Creality Ender-3) (Shenzhen, China) under the following conditions: temperature in the extruder of 220 °C, bed temperature of 60 °C, 0.12 mm between layers, smoothed fill pattern for the top infill, and monotonic fill pattern for the bottom infill using polylactic acid (PLA) as filling. For the thermal conductivity inside the reactor, two electrodes of 316L steel were added to the caps.

The biomass growth in the rice husk extract (40%) was filtered and weighed (1000 ppm); the wet biomass was added to a phosphate buffer in a range of pH 5.6–7.3. The samples were mixed and added into the 3D-printed reactor with a working volume of 20 mL. During the phycocyanin extraction, 40 V was applied in the reactor to reach the temperatures used in the experimental design (please see Table 2). The temperature control in the holding time was achieved manually by turning the power source on and off once the assay temperature was reached.

### 2.8. Experimental Design to Optimize the Phycocyanin Extraction by Ohmic Heating

Central compound rotatable design (CCRD) was applied. Temperature, time, and pH were studied as factors related to phycocyanin extraction and its optimization. The temperature applied was in the range of 30–80 °C. For the extraction of cyano-bacteria pigments, the temperature used in ohmic heating is 70 °C [40]; this temperature is related to a greater electroporation phenomenon and, consequently, permeation of the cell membrane for the release of pigments; lower temperatures in the 40–50 °C range are related to the extraction of phycocyanin [41]. The pH in the extraction of phycocyanin is in the range of above 5 and below 8, and the stability of these pigments is best in the range of 6–7 [42]. The time used for the extraction of cyanobacteria pigments is found in the range of 5–60 min [40,43]. Table 2 shows the levels for factors present in the CCRD with the coded levels. A total of 18 experimental runs were performed in random order. The response surface methodology (RSM) was used to optimize the studied factor from the second-order polynomic Equation (2).

The independent variables and levels (coded and un-coded) presented in Table 2 were used for the central compound design (Equation (2)).

(2) $$Y=\beta_{0}+\sum_{i=1}^{k}\beta_{i}X_{i}+\sum_{i<j}^{k}\beta_{ij}{X_{i}}^{2}+\sum \sum \beta_{ij}X_{i}X_{j}+\varepsilon$$

where Y represents the predicted response (phycocyanin content) and the independent variables correspond to X_1_, X_2_, and X_3_ [44]. The regression coefficients of variables for the intercept are as follows: β_0_ is a constant, β_i_ are linear coefficients, β_ii_ are quadratic coefficients, and β_ij_ are the coefficients of the interaction and *p* < 0.05. All the experimental assays were analyzed by the MATLAB r20217a software.

### 2.9. Statistical Analysis for the Evaluation of Rice Husk Extract as a Culture Medium

For the selection of the best treatment for the growth of *S. platensis* using rice husk as a culture medium and the analysis of the optimization of the phycocyanin extraction, a statistical analysis was applied. The analysis was performed using ANOVA followed by an analysis of the comparison of means by the Tukey method. All the analyses were performed considering 95% confidence and *p* ≤ 0.05. The software used was developed by Prof. Emilio Olivares-Sáenz at the School of Agronomy, Universidad Autónoma de Nuevo León (UANL, San Nicolás de las Garza, Mexico). The treatments used were duplicated using the mean of the replicates, with the standard deviation in the error bars.

## 3. Results

### 3.1. Rice Husk and Phycocyanin Composition Analysis by FTIR

**Figure 3:**
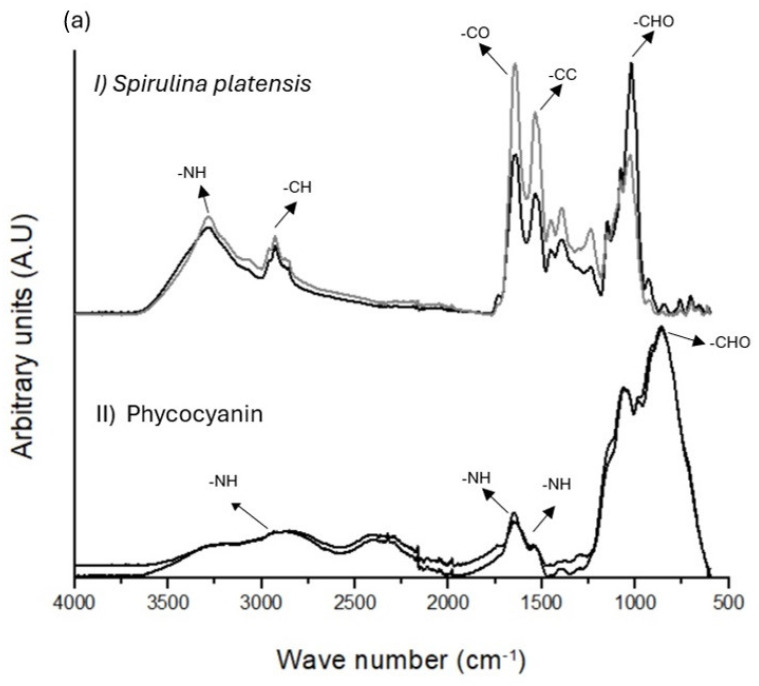

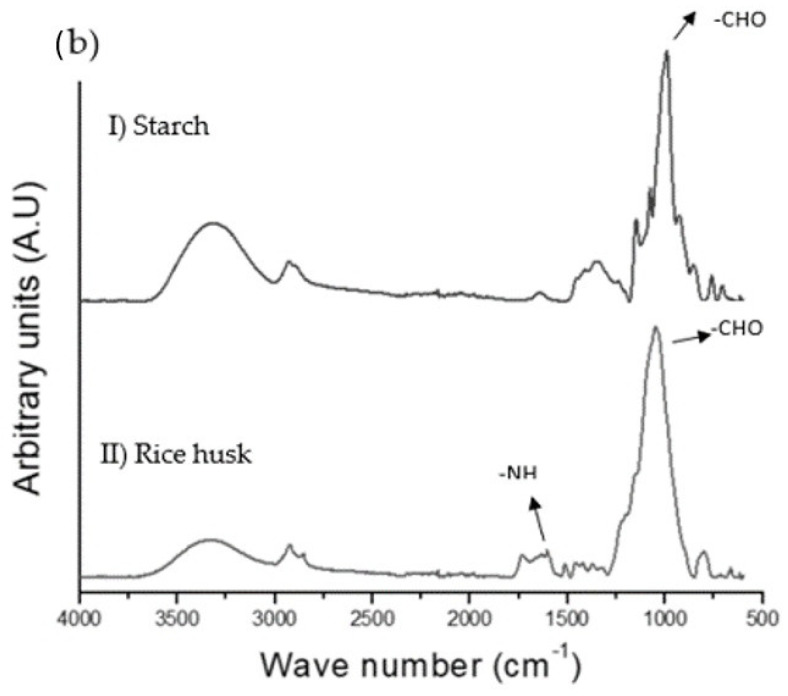
(**a**) FTIR analysis of the *S. platensis* biomass (II) and phycocyanin structure present in *S. platensis* biomass (II). (**b**) FTIR analysis of (I) the starch and (II) rice husk.

Fourier-transform analysis (FTIR) was used to detect the vibration of molecules present in the rice husk extract (carbohydrates, mainly starch) and phycocyanin (signals of amine I, II, and III). Please see Figure 3.

(a) I. The FTIR of the biomass of *S. platensis* presents different signals corresponding to different vibrations as follows: groups formed by stretching vibrations of -OH and -NH (3265 cm^−1^); stretching vibrations of -CH (2800–2900 cm^−1^); stretches of -CO corresponding to ketones, aldehydes mainly (1700–1400 cm^−1^); stretching vibrations of -C-O and -C-C (1300–1000 cm^−1^); and some signals corresponding to -*p*-O and -S-O (750–900 cm^−1^) [45]. *S. platensis* biomass shows a very similar composition; no evidence of difference is shown between the biomass obtained using the different cultures (rice husk extract and Zarrouk media).

II. The structural composition of phycocyanin remains consistent regardless of the growth conditions. This assertion applies consistently to phycocyanin extracted from biomass cultivated under control conditions (utilizing Zarrouk medium), as well as from biomass cultivated using rice husk extract. The composition of the phycocyanin corresponds to the following vibration signals: N-H amide I band (1640 cm^−1^), amide II (1500 cm^−1^), amide III (1460 cm^−1^), carbohydrates such as starch (1200 cm^−1^), and some signs of carbonates (700–850 cm^−1^) [46].

(b) I. Starch shows the typical trace of carbohydrates in the zones of 900–1200 cm^−1^ and 1200 cm^−1^ [37]. The rice husk presents signals in the carbohydrates zone (900–1200 cm^−1^), mainly starch, a protein composition with vibration signals of -NH close to 1500–1600 cm^−1^, and signals corresponding to silica (780–800 cm^−1^) [47]. The rice husk has components such as a source of nitrogen (presence of amine) and a source of carbohydrate (mainly starch) necessary for the growth of *S. platensis* biomass [38]. Enzymes commonly present in cyanobacteria are amylase, cellulases, and β-glucosidases; these types of enzymes could hydrolyze the glycosidic β bonds 1–4 of the chains of amylose and amylopectin present in the starch and could explain how *S. platensis* obtains glucose as a source of carbon. The presence of glucose can increase the yield of *S. platensis* biomass and increase the production of phycocyanin [48,49].

### 3.2. Ash Content and Elemental Composition of Rice Husk Extract by X-ray Fluorescence Spectrometry

The total solids content present in the rice husk extract was 0.90 ± 0.03 g/L. The elemental composition of rice husk extract (RHE) shows important compounds such as potassium at 22.48 ± 0.06%, silica at 1.95 ± 0.06%, magnesium at 2.17 ± 0.06%, phosphorous at 1.95 ± 0.06%, calcium at 1.83 ± 0.05%, iron at 0.03 ± 0.00%, and metal traces. The heating extraction of the components present in the rice husk extract was effective for the solubilization of nutrients necessary for the growth of *S. platensis.*

The presence of components such as nitrogen and a carbon source are essential for microalgae growth [48]. The trace metals are related to microalgae growth, especially the Fe^2+^, Cu^2+^, and Zn^2+^ present in the RHE. These metals are also associated with the production of pigments in microalgae [49]. NaNO_3_ has an important role in protein production, including that of phycobiliproteins. To produce phycocyanin, the presence of Fe^2+^ is fundamental; even though this element is not a structural part of phycocyanin, it is an important precursor of phycocyanin and co-factor in several enzymes. The enzyme ferrochelatase inserts a ferrous ion to produce a stable structure of heme. These structures are opened with an oxygenase enzyme, resulting in biliverdin IX α, a precursor of the phycobiliproteins [44].

### 3.3. Total Carbohydrates Present in Rice Husk Extract

The total carbohydrate content of the rice husk extract determined by the Anthrone method was 143 ± 0.07 mg/L, representing 31.8% ± 0.01 of the total solids. These carbohydrates in the rice husk are primarily composed of glucose and xylose.

### 3.4. Effect of the Concentrations of Rice Husk Extract on the Concentration of S. platensis Biomass

**Figure 4:**
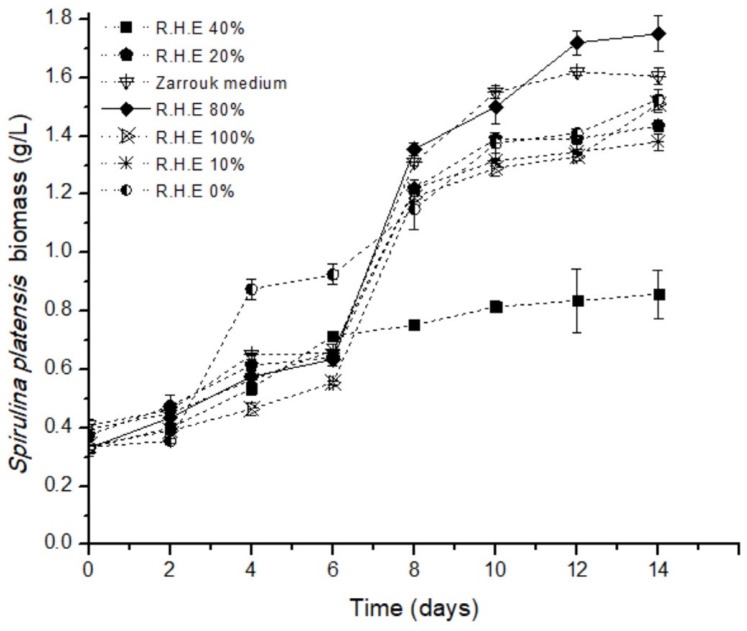
Effect of the different concentrations of rice husk extract (RHE) on the growth of *S. platensis* biomass, and the control Zarrouk’s medium.

Seven treatments were performed for the evaluation of the effect of the rice husk extract (RHE) on the concentration of the *S. platensis* biomass (carried out by duplicate); all the treatments had similar growth behavior, with marked growth on the eighth day (please see Figure 4). The range of biomass for the treatments of rice husk extract was 1.25–1.75 g/L. The highest concentration corresponds to treatment 4 (rice husk extract 40%) with a biomass content of 1.75 ± 0.71 g/L. The lowest yield corresponds to treatment 1 (0% rice husk) with a total yield of 0.9 ± 0.141 g/L. The treatments with rice husk show a better or equal yield in comparison with the Zarrouk medium; the control treatment has a content of biomass of 1.55 ± 0.03. When using Zarrouk as a culture medium, usually the biomass is in the range of 0.6–2.5 g/L, depending on the modified nutrients present in the Zarrouk medium [18]. The culture that contained different concentrations of rice husk extract and NaNO_3_ was efficient for the growth of *S. platensis* biomass, which is an alternative to growing microalgae. This is because the rice husk has nutrients that are present in the extract, such as sources of SiO_2_, sources of C (starch), and traces of elements such as Ca, Mg, Fe, and Zn, mainly [48,49]. At an appropriate pH of 7.5, all the present conditions promote the growth of *S. platensis* using rice husk extract as a culture medium.

### 3.5. Effect of the Concentrations of Rice Husk Extract on the Growth Rate, Protein Content, and Phycocyanin Content of S. platensis

To evaluate the effect of the rice husk extract as a culture medium, the protein content, phycocyanin content, and growth rate of *S. platensis* were analyzed. Table 3 shows the treatments analyzed. The highest specific growth rates are present in treatments 4 (40% *v*/*v* RHE) and 3 (20% *v*/*v*, RHE), with 0.125 ± 0.000 and 0.115 ± 0.001, respectively, followed by treatment 1 corresponding to Zarrouk medium. The use of rice husk as a culture medium proves to be suitable for the growth of *S. platensis* biomass compared to the synthetic Zarrouk medium.

The highest specific rates show that rice husk extract as a culture medium for *S. platensis* growth is better than the Zarrouk medium. The protein contents present in all the treatments show that the highest content is present in treatment 4, at 672.270 ± 0.260 mg/g, followed by treatment 1 corresponding to Zarrouk medium. The content of protein expected for *S. platensis* biomass in the literature is reported to be in the range of 600–700 mg/g [22].

Finally, phycocyanin is present in all the treatments. The highest phycocyanin content is 25–31 mg/g in treatments 2, 3, and 4, followed by treatment 1. The phycocyanin extracted by the freezing and thawing method is in the range of 19–73 mg/g of *S. platensis* biomass [50]. The use of rice husk extract as a culture medium for the growth of *S. platensis* is a viable method with some advantages such as easy preparation of the culture medium and lack of need to adjust the pH (7.6). The rice husk extract is an interesting alternative for the growth of *S. platensis* biomass; the composition of the different polysaccharides leads to the opportunity for the use of rice husk for the extraction of high-value compounds such as oligosaccharides and fermentable sugars to produce bioethanol for second-generation biorefineries, as well as culture media for microalgae.

### 3.6. Central Composite Rotatable Design Model for the Optimization of Phycocyanin Extraction Using Ohmic Heating

The effect of temperature, time, and pH on phycocyanin extraction using ohmic heating was investigated using CCRD. The levels of the independent variables used in the experimental design were determined based on preliminary experiments. Table 4 shows all the experimental runs (18), and the response (Y) of phycocyanin extraction was analyzed. The highest concentration of phycocyanin was extracted when the variables were in the central point as follows: pH 6.5, 10 min, and 55 °C. The highest concentrations of phycocyanin were in the range of 52–68 mg/g.

The linearity and quadratic effect of the treatments on the response variables were obtained by ANOVA and are shown in Table 5. The independent variables (temperature b2, time b3, and pH b4) are significant (*p* < 0.05). However, the interaction of the factors was not significant (*p* > 0.05), as is presented in Table 5. The mathematical model obtained from the coefficients in Table 5 has an R^2^ of 0.83 and a root mean square error (RMSE) of 12.6. Such values are acceptable for the extraction of phycocyanin by ohmic heating.

The predictive equation for the yield of phycocyanin (Equation (3)) is as follows:

Phycocyanin (mg/g) = 59.65 − 10.92 X_1_ − 4.93 X_2_ − 0.36 X_3_ − 12.75 X_1_^2^ − 9.55 X_2_^2^ − 14.59 X_3_^2^ − 0.25 X_1_X_3_ − 4.67 X_2_X_3_ (3)

**Figure 5:**
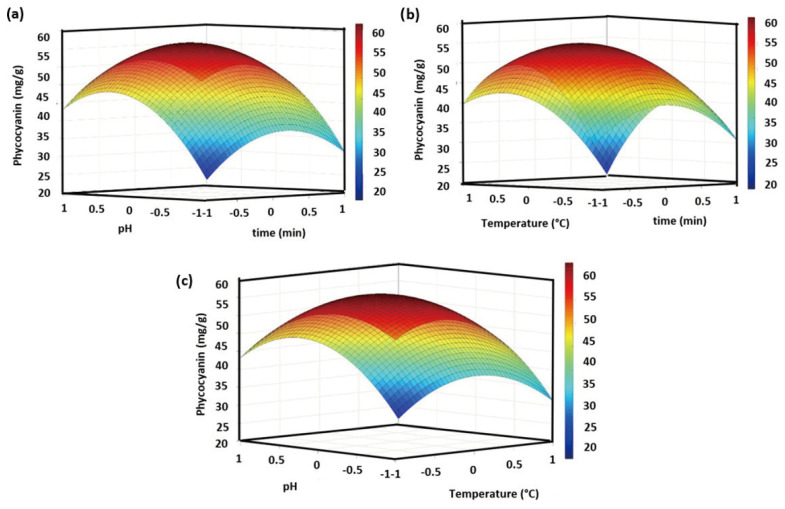
Effect of the variables (**a**–**c**) pH, time, and temperature on the ohmic heating for the content of phycocyanin present in the *S. platensis* biomass.

A three-dimensional response surface plot is presented in Figure 5. The effects of the variables on the extraction of phycocyanin are found in Figure 5a–c. To date, several reports exist on the use of ohmic heating for the extraction of different compounds; however, the implementation of these technologies for phycocyanin extraction has been limited. For the use of ohmic heating technology, the conditions reported were a temperature of 44 °C/ 30 min and 37 °C/60 min [40,50]. Under these operational conditions, the highest phycocyanin contents were 45 and 40 mg/g, respectively. In the present study, the experimental runs that were close to the reported values were runs 1, 3, 4, and 8. They present yields of phycocyanin in the range of 33–43 mg/g.

In runs 2, 3, and 8, similarities were found regarding high temperature values (≥40 °C) and the same time (10 min); however, the three runs presented high pH values of ≥7. A basic pH value can affect the stability of the pigment [45].

Although the phenomenon of electroporation and cellular permeability (due to heat and time) may be present, the pigment may have difficulties being released because there are no differences in the isoelectric point of phycocyanin (4.6–5.2) [46] and the phosphate buffer at ≥7 pH [45]. Experimental run 4 presents an adequate pH (6.5) and time (10 min); however, the low temperature (≤40 °C) presented difficulty for the electroporation phenomenon and membrane permeability. It is important to mention that the temperature values of experimental runs 3, 4, and 8 are very similar to those reported in the literature [39,50].

This indicates that low temperatures (≤40 °C) are inadequate for achieving higher yields of phycocyanin. Experimental runs 6, 9, and 15 were exposed to a high temperature of 70 °C. Under this temperature, although membrane permeability and electroporation are favored, pigment degradation was observed, with a yield close to 7 mg/g. These treatments obtained the lowest amounts of pigment and lost the characteristic blue color of phycocyanin.

For the highest content of phycocyanin, the variables are close to the central points, as follows: time of 10 min, temperature of 55 °C, and pH of 6.5. The highest concentrations of phycocyanin are in the range of 52–68 mg/g.

The content of phycocyanin increased when using the optimization process, reaching a total of 75.80 ± 0.98 mg/g. The results indicate that under these conditions, it is possible to extract a greater amount of phycocyanin. The predictive equation indicated that the expected content of phycocyanin was 62.54 mg/g. The difference between the theoretical and actual values is mainly due to the adjustment of the model used.

### 3.7. Optimization of the Extraction of Phycocyanin Using Ohmic Heating

The independent variables influence the extraction of phycocyanin present in the *S. platensis* biomass (*p* < 0.05). The predicted model showed an optimal concentration of phycocyanin of 62.64 (mg/g) when the temperature, time, and pH were 50.71 °C, 8.60 min, and 6.52, respectively. However, when the phycocyanin concentration was evaluated, the optimal yield was 75.79 ± 0.98 (mg/g).

The results present in the experimental design showed a higher concentration of phycocyanin in comparison with the predicted model. The recovery yield of phycocyanin present in the ohmic heating process is higher in comparison with the freezing and thawing method using rice husk extract as a culture medium in both cases, with the freezing and thawing method showing a phycocyanin concentration in the range of 25–31 mg/g. The optimal conditions of the CCDR showed that extraction needs to be carried out at 50 °C. To maintain the structure of the proteins present, it is recommended that the temperature does not exceed 60 °C, as higher temperatures have been reported to denature proteins. It is important to mention that the exposure time at 50 °C in this study was separated into short extraction periods (8 min), so critical temperatures were not reached [47]. Temperatures above 60 °C can denature the secondary, tertiary, and quaternary structures of allophycocyanin, affecting the color and stability of phycocyanin [18,50].

The temperature used for the phycocyanin extraction showed an impact on the cell wall membrane, and the electroporation under these conditions was higher in comparison with that at lower temperatures; the consequence of membrane permeability causes the release of the pigments present in the biomass of *S. platensis*. The appropriate temperature for this purpose is 50 °C, and factors such as a pH of 6.5 are essential for the ionic interaction of phycocyanin and the buffer related to the release of this protein pigment. On the other hand, ohmic heating processing is a promising alternative to use at other scales of operation in the extraction of compounds. However, scaling-up strategies need to be studied. Furthermore, several advantages can be attributed to this technology, such as the construction materials for the reactor and the possible energy consumption, which, by using relatively low temperatures, could be low. Finally, phycocyanin extraction showed good yields compared to conventional methods; electroporation and permeabilization of the *S. platensis* membrane under optimized conditions allowed the release of phycocyanin into the liquid medium. Furthermore, the ohmic heating process is an alternative for the release of other intracellular compounds, not only phycocyanin, in short periods of operation.

## 4. Conclusions

This work showed the development of a bioprocess for the cultivation and growth of *S. platensis* in an alternative medium such as rice husk extract. The use of this agro-industrial waste allows the integration of the second and third generation of biorefineries. In addition, the design and construction of a 3D-printed reactor was efficient in the extraction of phycocyanin from the cultivated *S. platensis* and had good performance under the operating conditions studied. Furthermore, the electroporation phenomenon in addition to membrane permeability allows compounds to be extracted without altering their properties, as they are released into the medium (buffer) through ionic interaction under the study conditions present in ohmic heating. This development will provide the basis for scaling up the process and obtaining compounds of industrial interest, such as phycocyanin, under a circular bioeconomy concept and incorporating them into a food matrix.

## Figures and Tables

**Table 1:** Treatments with different concentrations of rice husk as a culture medium for the growth of *S. platensis*.

Treatments	Rice Husk Extract */Medium (%, *v*/*v*)	Distilled Water (%, *v*/*v*)
T1	0	100
T2	10	90
T3	20	80
T4	40	60
T5	80	20
T6	100	0
T7 (blank)	Zarrouk	NA

* Initial concentration 40 g/L; NA = not applicable.

**Table 2:** Coded levels and variables of the CCDR for experimental design.

Variables	Symbols	−1.68 (α)	−1	0	+1	1.68 (α)
Temperature (°C)	X_1_	29.77	40	55	70	80.23
Time (min)	X_2_	1.59	50	10	15	18.41
pH	X_3_	5.66	6	6.5	7	7.34

**Table 3:** Effect of the rice husk extract (RHE) on the specific growth, protein content, and phycocyanin content in the biomass of *S. platensis*.

Treatments/Rice Husk (%)	Specific Growth Rate µ (h^−1^)	Protein Content (mg/g)	Phycocyanin Content (mg/g)
T1 Zarrouk	0.109 ± 0.003 ^c^	608.940 ± 12.720 ^b^	25.25 ± 2.600 ^ab^
T2 R.H 10	0.107 ± 0.002 ^e^	499.000± 24.030 ^c^	31.46 ± 0.750 ^a^
T3 R.H 20	0.115 ± 0.001 ^b^	544.550± 22.690 ^c^	29.31 ± 0.240 ^a^
T4 R.H 40	0.125 ± 0.000 ^a^	672.270 ± 0.260 ^a^	28.47 ± 3.040 ^a^
T5 R.H 80	0.108 ± 0.002 ^d^	502.330 ± 8.810 ^c^	21.55 ± 1.890 ^bc^
T6 R.H 100	0.099 ± 0.002 ^f^	591.220 ± 17.100 ^b^	23.24 ± 0.260 ^c^
T7 Water *	0.055 ± 0.038 ^g^	326.770 ± 17.100 ^d^	19.83 ± 6.380 ^ab^

Notes: values are presented as the means ± standard deviations. Values in the same column with different letters are significantly different (Tukey’s analysis, *p* ≤ 0.05). * RH (0%) = water = control.

**Table 4:** Effects of the different experimental runs on phycocyanin extraction.

Run	X_1_	X_2_	X_3_	Phycocyanin (mg/g)
1	0	0	0	64.70
2	0	0	−1.68	33.70
3	−1	−1	1	39.80
4	−1.68	0	0	44.20
5	0	0	0	68.40
6	1.68	0	0	7.90
7	−1	−1	−1	24.30
8	−1	1	1	43.10
9	1	−1	−1	7.70
10	−1	1	−1	20.80
11	0	0	0	52.50
12	0	−1.68	0	55.00
13	1	−1	1	12.30
14	1	1	−1	12.00
15	1	1	1	7.80
16	0	0	1.68	8.00
17	0	0	0	52.15
18	0	1.68	0	15.20

Coded variables: X_1_ = temperature (°C), X_2_ = time (min), X_3_ = (pH).

**Table 5:** Analysis of variance (ANOVA) of the second-order polynomial model for the extraction of phycocyanin using ohmic heating.

Source	Estimated	Stnd. Err	tStat	*p*-Value
b1	59.700	6.257	9.541	0.000
b2	−21.915	6.826	−3.210	0.012
b3	−9.741	6.938	−1.403	0.197
b4	−28.787	7.041	−4.088	0.003
b5	−25.494	7.134	−3.573	0.007
b6	−20.710	7.617	−2.718	0.02
b7	−0.724	6.803	−0.106	0.917
b8	0.016	8.885	0.001	0.99
b9	−9.344	8.885	−1.05	0.323
b10	−0.513	8.885	−0.057	0.955

## Data Availability

The original contributions presented in the study are included in the article, further inquiries can be directed to the corresponding author.
